# Deoxygenation enhances photosynthetic performance and increases N_2_ fixation in the marine cyanobacterium *Trichodesmium* under elevated *p*CO_2_

**DOI:** 10.3389/fmicb.2023.1102909

**Published:** 2023-02-15

**Authors:** He Li, Kunshan Gao

**Affiliations:** ^1^State Key Laboratory of Marine Environmental Science, College of Ocean and Earth Sciences, Xiamen University, Xiamen, China; ^2^Co-Innovation Center of Jiangsu Marine Bio-industry Technology, Jiangsu Ocean University, Lianyungang, China

**Keywords:** growth rate, N_2_ fixation, photosynthesis, respiration, *Trichodesmium erythraeum*

## Abstract

Effects of changed levels of dissolved O_2_ and CO_2_ on marine primary producers are of general concern with respect to ecological effects of ongoing ocean deoxygenation and acidification as well as upwelled seawaters. We investigated the response of the diazotroph *Trichodesmium erythraeum* IMS 101 after it had acclimated to lowered *p*O_2_ (~60 μM O_2_) and/or elevated *p*CO_2_ levels (HC, ~32 μM CO_2_) for about 20 generations. Our results showed that reduced O_2_ levels decreased dark respiration significantly, and increased the net photosynthetic rate by 66 and 89% under the ambient (AC, ~13 μM CO_2_) and the HC, respectively. The reduced *p*O_2_ enhanced the N_2_ fixation rate by ~139% under AC and only by 44% under HC, respectively. The N_2_ fixation quotient, the ratio of N_2_ fixed per O_2_ evolved, increased by 143% when *p*O_2_ decreased by 75% under the elevated *p*CO_2_. Meanwhile, particulate organic carbon and nitrogen quota increased simultaneously under reduced O_2_ levels, regardless of the *p*CO_2_ treatments. Nevertheless, changed levels of O_2_ and CO_2_ did not bring about significant changes in the specific growth rate of the diazotroph. Such inconsistency was attributed to the daytime positive and nighttime negative effects of both lowered *p*O_2_ and elevated *p*CO_2_ on the energy supply for growth. Our results suggest that *Trichodesmium* decrease its dark respiration by 5% and increase its N_2_-fixation by 49% and N_2_-fixation quotient by 30% under future ocean deoxygenation and acidification with 16% decline of *p*O_2_ and 138% rise of *p*CO_2_ by the end of this century.

## 1. Introduction

Biological N_2_ fixation by marine diazotrophs is an important source of “new” usable nitrogen to phytoplankton in oligotrophic surface waters (Karl et al., 1997; Bergman et al., 2013). The N_2_-fixing cyanobacteria, *Trichodesmium* spp., contribute about half of the current estimate of global annual marine N_2_ fixation, playing a pivotal role in global biogeochemical N/C cycles (Mahaffey et al., 2005; Sohm et al., 2011). The nitrogenase enzyme encoded by the *nif*HDK genes is critically sensitive to and can be inactivated by oxygen (O_2_) (Staal et al., 2007; Hutchins and Sañudo-Wilhelmy, 2022). Most cyanobacterial N_2_-fixers can avoid O_2_ damage *via* temporal (fixing N_2_ during night) or spatial (using heterocyst) strategies. However, *Trichodesmium* spp. do not possess such contrivances to avoid photosynthetically evolved O_2_, since all cells of the trichomes have PSII (Lin et al., 1998; Bergman et al., 2013). It is known that *Trichodesmium* cells down-regulate its photosynthesis during midday to protect nitrogenase using various physiological pathways (e.g., the Mehler reaction, respiration and flavoprotein-mediated O_2_ uptake) to sequestrate O_2_ under light (Berman-Frank et al., 2001; Gallon, 2001; Milligan et al., 2007), which is considered as a metabolic strategy for the O_2_-sensitive diazotroph (Luo et al., 2022).

Dissolved O_2_ (DO) levels in the oceans have been declining due to global warming, and are expected to further decline during the 21th century (Schmidtko et al., 2017; Breitburg et al., 2018). Rising global temperatures decrease the solubility of O_2_, enhancing stratification (hindering ventilation to deeper layers) of the upper mixing layers and increasing biological respiration, thus ultimately reduce DO in surface and subsurface oceans (Carstensen et al., 2014). Consequently, the oxygen-minimum zones (OMZ) have expanded horizontally and vertically along with progressive ocean deoxygenation (Stramma et al., 2008; Schmidtko et al., 2017). On the other hand, internal O_2_ concentrations of *Trichodesmium* colonies or blooms ranged from 0 to 500 μM during dark and/or high-light periods, which is supposed to affect the nitrogenase activity (Paerl and Bebout, 1992; Eichner et al., 2019). Light stimulation of nitrogenase activity is most obvious at low O_2_ concentrations, while it becomes gradually less important with increased levels of O_2_ in *Trichodesmium* IMS 101 (Staal et al., 2007). Therefore, it is likely that the marine diazotrophs including *Trichodesmium* would benefit from ocean deoxygenation.

On the other hand, ocean acidification (OA) induced by continuous dissolution of anthropogenically emitted CO_2_ into seawater is known to affect various phytoplankton species and ecological processes (Gao et al., 2020), which show positive and/or negative responses to OA under different environmental conditions (Boyd et al., 2018). Temperature, light, nutrients and UV radiation are known to modulate the influences of elevated *p*CO_2_, resulting in negative in oligotrophic waters but in neutral or positive effects in nutrients-replete waters (Gao et al., 2022). Most cyanobacteria and microalgae, including *Trichodesmium*, have evolved efficient CO_2_-concentrating mechanisms (CCMs) that actively uptake CO_2_ and bicarbonate to increase the level of intracellular dissolved inorganic carbon for efficient photosynthesis (Giordano et al., 2005). Therefore, the energy saved by the downregulation of CCMs under elevated *p*CO_2_ is expected to be reallocated to other metabolic processes (Kranz et al., 2010). Differential responses have also been documented in *Trichodesmium*. OA has been shown to promote the N_2_ fixation and growth of *Trichodesmium* (Hutchins et al., 2007; Levitan et al., 2007; Garcia et al., 2011; Hutchins et al., 2013). However, it was also shown to result in insignificant or even negative effects on *Trichodesmium*, and the negative effects were more obvious under iron limitation (Shi et al., 2012; Hong et al., 2017; Zhang et al., 2019). It was suggested that the energy saved from downregulation of CCMs under elevated *p*CO_2_ is not enough for the energy demand of increased N_2_-fixation rates in *Trichodesmium* under OA conditions (Luo et al., 2019). While these disputable findings need to be mechanistically reconciled, effects of multiple drivers on the marine diazotroph should also be examined (Wake, 2019). Recently, it was shown that reduced O_2_ availability modulated the effects of elevated *p*CO_2_ on a diatom (Sun et al., 2022). Combined effects of ocean deoxygenation and acidification on *Trichodesmium* has yet to be investigated.

Since levels of pH/*p*CO_2_ and O_2_ in the center of *Trichodesmium* colonies fluctuate during a diel cycle due to night respiration and daytime photosynthesis (Eichner et al., 2019), its physiological performance can be naturally influenced by the changed levels of both O_2_ and CO_2_. In parallel, progressive ocean deoxygenation and acidification are covarying factors that affect marine organisms including the diazotrophs (Staal et al., 2007; Paulmier et al., 2011; Sun et al., 2022). Considering that low levels of O_2_ can theoretically enhance carboxylation *via* decreasing oxygenation processes catalyzed by Rubisco and stimulate the activity of nitrogenase, we hypothesize that combination of ocean deoxygenation and acidification can synergistically enhance photosynthetic performance and N_2_ fixation of *Trichodesmium*. Hence, we simulated different microenvironments by growing *Trichodesmium erythraeum* (*T. erythraeum*) strain IMS 101 under four different *p*O_2_/*p*CO_2_ combinations (ambient CO_2_ & ambient O_2_; ambient CO_2_ & low O_2_; high CO_2_ & ambient O_2_; high CO_2_ & low O_2_) in order to investigate the responses of *Trichodesmium*. In this work, we found that reduced O_2_ concentration increased both photosynthetic performance and N_2_ fixation rate and thus raised the particulate organic carbon and nitrogen even under elevated *p*CO_2_ projected for future ocean acidification by the end of this century.

## 2. Materials and methods

### 2.1. Culture conditions

*Trichodesmium erythraeum* (strain IMS 101), originally isolated from the North Atlantic Ocean (Prufert-Bebout et al., 1993), was grown in YBCII medium without combined N prepared with autoclaved sterilized artificial seawater (Chen et al., 1996). Experiments were conducted in polycarbonate bottles under 160 μmol photons m^−2^ s^−1^ of PAR (measured by a Solar light sensor, PMA2100, United States) with a day-night cycle of 12: 12 h at 27 ± 0.5°C in an incubator (HP300G–C, Ruihua, China).

Triplicate experiments were carried out in a four treatments matrix of two levels of CO_2_ (ambient and high CO_2_) and two levels of O_2_ (ambient and low O_2_), respectively. *T. erythraeum* cells were maintained in the exponential growth phase by semicontinuous culture (diluted every 48 h). The chlorophyll *a* (Chl *a*) concentration was maintained within a range of 0.003–0.015 μg mL^−1^ (Supplementary Figure S1), so that dissolved O_2_ and carbonate chemistry in the cultures were little altered. The carbonate chemistry parameters were relatively stable before and after the dilution, the pH variations were less than 0.08 units under either the HC (elevated CO_2_, 32 μM) or AC (ambient CO_2_, 12 μM) treatments, along with 12 to 19% decrease of *p*CO_2_ before the dilutions, and dissolved O_2_ concentrations varied less than 10 μM (245–255 μM) and 30 μM (60–90 μM) under AO (ambient O_2_) and LO (low O_2_) treatments, respectively (Table 1; Supplementary Figure S2). Prior to dilution, fresh media were prepared with the target O_2_ and CO_2_ levels: ambient CO_2_ & ambient O_2_ (ACAO), ambient CO_2_ & low O_2_ (ACLO,), high CO_2_ & ambient O_2_ (HCAO), high CO_2_ & low O_2_ (HCLO) (Table 1). A customized CO_2_/O_2_ controlling device (CE-100DY, Ruihua, China) was employed to achieve the above O_2_ and CO_2_ levels. The bottles were shaken gently every 3 h during the daytime to ensure that the cyanobacterial filaments were in a suspended state.

**Table 1:** Dissolved O_2_ concentrations and carbonate chemistry parameters of the culture media during the semi-continuous cultures of *Trichodesmium erythraeum* IMS101 under the four treatments (ACAO, ~12  **μ**M CO_2_/~250   **μ**M O_2_; ACLO, ~12  **μ**M CO_2_/~60   **μ**M O_2_; HCAO, ~32   **μ**M CO_2_/~250   **μ**M O_2_; HCLO, ~32   **μ**M CO_2_/~60   **μ**M O_2_).

			O_2_ (μM)	pH_NBS_	TA (μM)	DIC (μM)	CO_2_ (μM)	HCO_3_^−^ (μM)	CO_3_^−^ (μM)
A	AC	AO	248.7 ± 1.5^a^	8.17 ± 0.00^a^	2368.9 ± 32.8^a^	2042.7 ± 30.1^a^	12.14 ± 0.19^ac^	1825.9 ± 27.1^a^	204.7 ± 2.9^a^
		LO	60.2 ± 0.8^b^	8.15 ± 0.01^a^	2365.5 ± 19.3^a^	2047.5 ± 18.5^a^	12.60 ± 0.32^a^	1835.5 ± 17.8^a^	199.4 ± 3.5^a^
	HC	AO	245.0 ± 1.0^a^	7.79 ± 0.01^b^	2373.5 ± 19.1^a^	2222.7 ± 17.1^b^	32.74 ± 0.32^b^	2090.4 ± 15.6^b^	99.5 ± 1.8^b^
		LO	61.9 ± 1.0^b^	7.81 ± 0.01^b^	2390.1 ± 18.3^a^	2232.0 ± 19.1^b^	31.56 ± 0.73^b^	2096.6 ± 18.7^b^	103.9 ± 1.3^b^
B	AC	AO	256.7 ± 1.5^c^	8.20 ± 0.01^c^	2394.1 ± 12.0^a^	2042.9 ± 17.5^a^	11.02 ± 0.41^c^	1810.2 ± 20.3^a^	221.7 ± 3.2^c^
		LO	89.2 ± 1.6^d^	8.21 ± .01^c^	2402.0 ± 36.4^a^	2046.0 ± 29.9^a^	10.85 ± 0.08^c^	1810.0 ± 24.3^a^	225.1 ± 5.7^c^
	HC	AO	253.3 ± 1.5^c^	7.86 ± 0.02^d^	2350.9 ± 55.4^a^	2173.4 ± 46.4^b^	27.16 ± 0.56^d^	2032.7 ± 40.4^b^	113.5 ± 6.6^bd^
		LO	91.3 ± 2.8^d^	7.90 ± 0.01^d^	2409.2 ± 17.0^a^	2212.5 ± 17.8^b^	25.10 ± 0.50^e^	2061.2 ± 17.4^b^	126.2 ± 1.2^e^

Parameters were estimated after (A) and before (B) dilutions of the cultures every 48 h. The values of pH_NBS_ and total alkalinity (TA) were measured and other carbonate chemistry parameters were obtained accordingly by using the CO2SYS. Values are means ± SD of triplicate cultures. Different superscripted letters indicate significant (*p* < 0.05) differences among the treatments.

### 2.2. Carbonate chemistry and dissolved O_2_ parameter in cultures

The dissolved O_2_ and pH of seawater were measured before and after dilution every 2 days. The dissolved O_2_ was measured with a Clark-type oxygen electrode (Hansatech, United Kingdom). The pH_NBS_ was measured using a pH meter, which was three-point calibrated with NBS buffer. Total alkalinity (TA) was measured using the Gran potentiometric titration method (Gao et al., 2018). Dissolved inorganic carbon (DIC) and other carbonate chemistry parameters (Table 1) were calculated from pH_NBS_ and TA by using the CO2SYS software (Lewis et al., 1998).

### 2.3. Chl *a* concentration and specific growth rate

*Trichodesmium erythraeum* cells were gently filtered onto 25 mm glass-fiber filters (GF/F, Whatman), which was then extracted in pure methanol at 4°C for 24 h for Chl *a* determination. The extracts were centrifuged at 6000 g for 10 min, and the absorbances of the supernatants at 665 nm and 750 nm were measured using a UV–VIS Spectrophotometer (DU800, Beckman, United States). The Chl *a* concentration was calculated according to the equations Chl *a* (μg mL^−1^) =12.9447 × (A_665_ − A_750_) × V1/V2 (Ritchie, 2006), where V1 and V2 represent the 100% methanol volume (mL) and filtered cell volume (mL), respectively. The specific growth rates (μ, d^−1^) were determined from linear regressions of the natural log of Chl *a* vs. time during the exponential growth (Yi et al., 2020).

### 2.4. Dark respiration and net photosynthetic oxygen evolution

Dark respiration and net photosynthetic oxygen evolution were measured in the middle of the light period within 3 h using a Clarke-type electrode (Hansatech, United Kingdom). Cells were harvested by filtering onto polycarbonate membrane filters (5 μm, Millipore, Germany) under gentle vacuum pressure (<0.01 MPa). These cells were then re-suspended in seawater buffered with 20 mM Tris–HCl with a final Chl *a* concentration of approximately 0.5 μg mL^−1^. The pH levels of the Tris buffered-medium were pre-adjusted by adding hydrochloric acid or sodium hydroxide to the same levels of the cultures (pH 7.83 for HC and 8.13 for AC), and the O_2_ levels were achieved by flushing the medium with pure N_2_. The resuspended cells were injected into an oxygen electrode vessel with a magnetic stirrer held in a water-jacked chamber (temperature controlled at 27°C) under the same level of light intensity. The respiration rate was estimated in darkness by covering the reaction chamber with aluminum foil. Photosynthetic O_2_ evolution was determined under growth O_2_ levels.

### 2.5. Nitrogen fixation rate

N_2_-fixation rates were determined at the midpoint of the photoperiod with the acetylene reduction assay, assuming a ratio of 4: 1 to convert ethylene production to N_2_ fixation (Capone, 1993). Although one time point measurement has been able to identify changes of N_2_-fixation rate under different environmental conditions (Zhang et al., 2019), it may overlook other timepoint values of the N_2_-fixation during the whole light period. For determination of the N_2_-fixation rates, 5 ml of sample was added to a 13 mL vial, which was then sealed. After 1 mL of air was extracted from the headspace, 1 ml of acetylene (C_2_H_2_) was added to each vial. All replicates were mixed well and incubated for 2 h under the same temperature and light in the incubator as the growth conditions. The ethylene production was measured using a gas chromatograph with a flame ionization detector (Clarus 580, PerkinElmer, United States).

### 2.6. Particulate organic carbon and nitrogen (PON)

Cell samples for particulate organic carbon (POC) and particulate organic nitrogen (PON) were collected onto pre-combusted (450°C for 6 h) GF/F filters (Whatman), and were stored at −80°C before measuring. Filters were exposed to HCl fumes to remove inorganic carbon and dried at 60°C before analysis with a CHNS elemental analyzer (vario EL cube, Elementar, Germany).

### 2.7. Statistical analysis

The data were presented as means of three replicates ± SD (*n* = 3, triplicate independent cultures). One-way ANOVA and Tukey test were performed to analyze the statistical differences among different treatments. Differences were termed significant when *p* < 0.05. Before performing parametric tests, data were tested for homogeneity of variance (Levene test) and normality (ShapiroWilk test).

## 3. Results

### 3.1. Specific growth rate

**Figure 1:**
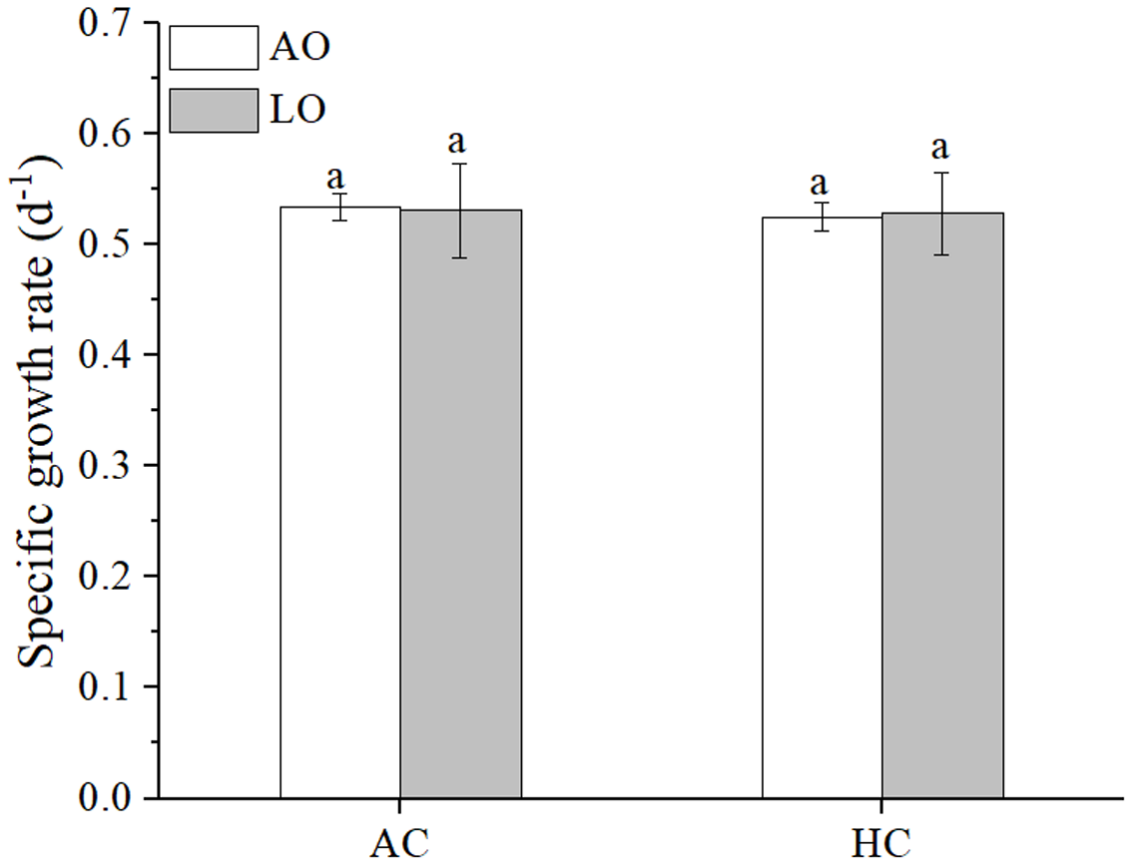
The specific growth rates of *Trichodesmium*. *erythraeum* IMS101 grown and measured after it acclimated for 16 days (12 generations) to different combinations of *p*CO_2_ and *p*O_2_ (AO, ~245 μM; LO, ~60 μM; AC, ~12 μM; HC, ~32 μM. LO and HC refer to lowered *p*O_2_ and elevated *p*CO_2_). The values are indicated as the means ± SD for independent cultures (*n* = 6) at each treatment. The same letters above the bars indicate no significant (*p* < 0.05) differences among the treatments.

Neither elevated *p*CO_2_ (*p* = 0.9881) nor lowered *p*O_2_ (*p* = 0.6284) significantly affected the specific growth rate of *T. erythraeum* (Figure 1). The specific growth rates were 0.534 ± 0.012 and 0.531 ± 0.043 d^−1^ under AC, and were 0.525 ± 0.013 and 0.528 ± 0.038 d^−1^ under HC, at the levels of ambient and lowered O_2_, respectively (Figure 1).

### 3.2. Photosynthetic performance

**Figure 2:**
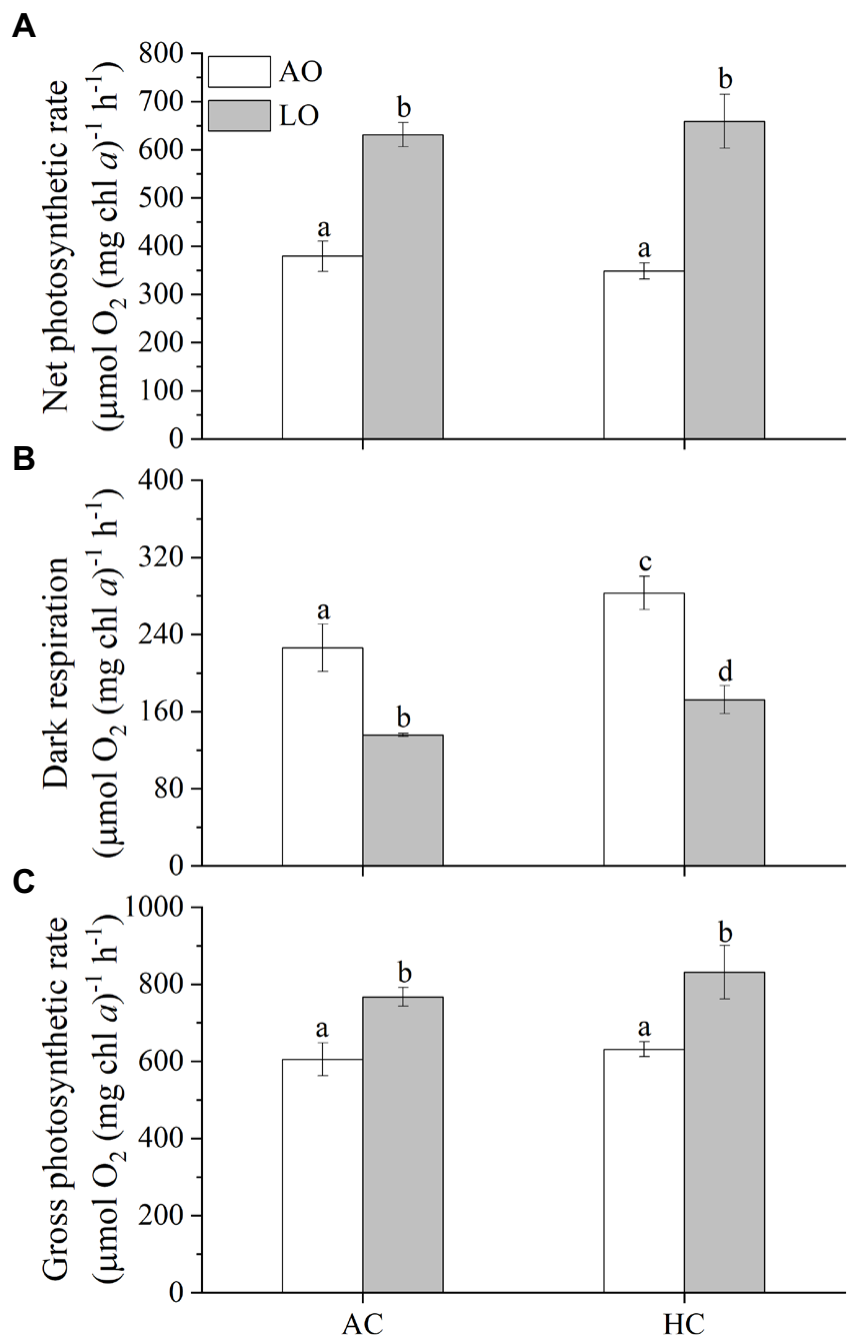
The rates of net photosynthetic O_2_ evolution **(A)**, dark respiration **(B)** and gross photosynthetic O_2_ evolution **(C)** in *T*. *erythraeum* IMS101 cells after acclimated different levels of *p*CO_2_ and *p*O_2_ (as shown in Figure 1) for 28–30 days (21–23 generations). Levels of *p*CO_2_ and *p*O_2_ are the same or within the same ranges as shown in Figure 1. The values are indicated as the means ± SD for triplicate cultures at each treatment. Different letters above the bars indicate significant (*p* < 0.05) differences among the treatments.

Dark respiration decreased with lowered levels of O_2_, whereas net and gross photosynthetic rate increased (Figure 2). Reduced O_2_ levels (from 250 to 60 μM) significantly increased net photosynthetic rate by 66% under AC (631 vs. 379 μmol O_2_ mg chl *a*^−1^ h^−1^, *p* < 0.0001) and by 89% under HC conditions (659 vs. 348 μmol O_2_ mg chl *a*^−1^ h^−1^, *p* < 0.0001), respectively (Figure 2A). Nevertheless, no significant differences were found in the net photosynthetic rate between the AC and HC treatments (*p* = 0.9432) (Figure 2A), though the mean net photosynthetic rate of the cells grown under HC was higher by about 4% than that of AC under LO. Decreased O_2_ levels to 60 μM (by 75%) significantly inhibited dark respiration by 40% under the AC (136 vs. 226 μmol O_2_ mg chl *a*^−1^ h^−1^, *p* = 0.0002) and 39% under the HC (172 vs. 283 μmol O_2_ mg chl *a*^−1^ h^−1^, *p* < 0.0001), respectively (Figure 2B), reflecting that *Trichodesmium* would decrease its dark respiration by 9% and increase its net photosynthetic rate by 14% with 16% *p*O_2_ decline by the end of this century (Table S1). Meanwhile, the HC-acclimated cells had higher (by 25% at AO and by 27% at LO) dark respiration rate compared to that of AC cells regardless of O_2_ levels (*p* = 0.0031 for AO, *p* = 0.0278 for LO). The reduced O_2_ levels significantly increased gross photosynthetic rate by 27% (767 vs. 605 μmol O_2_ mg chl *a*^−1^ h^−1^, *p* < 0.0019) under AC and by 32% (831 vs. 631 μmol O_2_ mg chl *a*^−1^ h^−1^, *p* = 0.0005) under HC, respectively (Figure 2C). Meanwhile, HC treatments increased gross photosynthetic rate by 4% (*p* = 0.4844) under AO and by 8% (*p* = 0.1081) under LO compared to the AC-grown cells, though the difference was insignificant.

### 3.3. Nitrogen fixation rate

**Figure 3:**
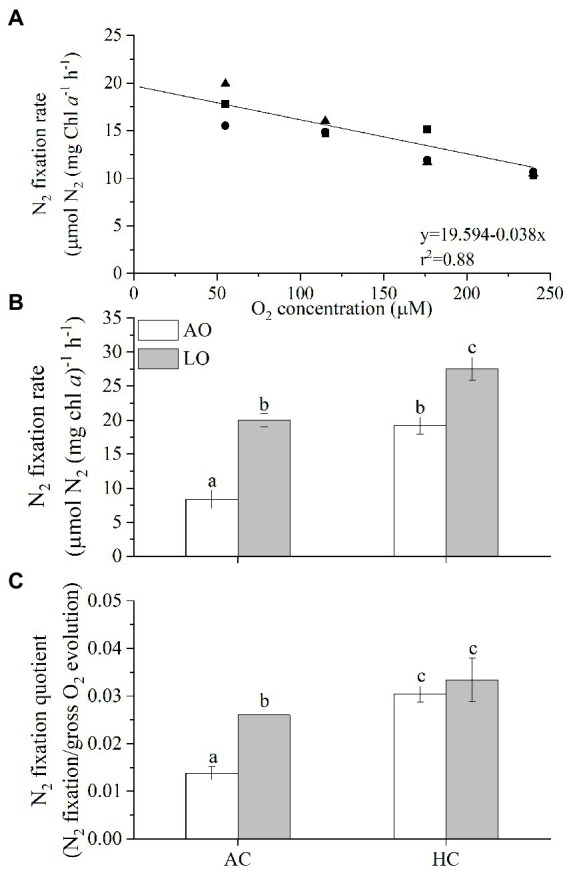
**(A)** N_2_-fixation rates of *T*. *erythraeum* IMS101 as a function of dissolved O_2_ concentrations in cultures after the cells acclimated for 48 h, **(B)** the N_2_-fixation rates and **(C)** N_2_-fixation quotients of *T*. *erythraeum* cells after acclimated different levels of O_2_ and CO_2_ (see Figure 1) for 32 days (24 generations). Different symbols [panel **(A)**] represent independent cultures (*n* = 3). The values in panels **(B,C)** indicate the means ± SD for triplicate cultures at each treatment. Levels of *p*CO_2_ and *p*O_2_ are the same or within the same ranges as shown in Figure 1. Different letters above the bars indicate significant (*p* < 0.05) differences among the treatments.

When *T. erythraeum* cells were grown under different O_2_ levels, their N_2_ fixation rates showed a negative relationship with the O_2_ concentrations (Figure 3A, r^2^ = 0.88). The N_2_ fixation rate increased linearly by 9% with each 10% decline in the dissolved O_2_ concentration. After the *Trichodesmium* cells acclimated to different levels of O_2_ and *p*CO_2_, lowering DO to about 60 μM increased the Chl *a*-specific N_2_ fixation rate by ~139% (20.0 vs. 8.4 μmol N_2_ mg chl *a*^−1^ h^−1^, *p* < 0.0001) under AC but only by 44% (27.5 vs. 19.2 μmol N_2_ mg chl *a*^−1^ h^−1^, *p* < 0.0001) under the HC conditions (Figure 3B), indicating obvious less enhancement by the reduced O_2_ treatment under the elevated *p*CO_2_. The *Trichodesmium* cells grown in the HC treatment had higher N_2_ fixation rates than those grown in the AC cultures regardless of the O_2_ levels (*p* < 0.0001 for AO, *p* = 0.0001 for LO), being increased by 129% under AO and by 38% under LO, respectively. The ratio of N_2_ fixation to the gross photosynthetic rate, as a proxy of the N_2_-fixation quotient (NFQ), ranged from 0.014 to 0.033 (mol N_2_: mol O_2_) (Figure 3C). The lowered *p*O_2_ treatment increased the NFQ by 89% under AC (*p* = 0.0016) and by only 10% under HC (*p* = 0.5104), and the elevated *p*CO_2_ and low *p*O_2_ treatment increased it by 143% compared to ACAO (*p* < 0.0001). Based on the results in this work, we estimated that *Trichodesmium* would increase its N_2_-fixation by 14% and N_2_-fixation quotient by 19% under future ocean deoxygenation with 16% decline of *p*O_2_ till 2,100 under the current *p*CO_2_ level (Table S1).

### 3.4. Particulate C and N quotas

**Figure 4:**
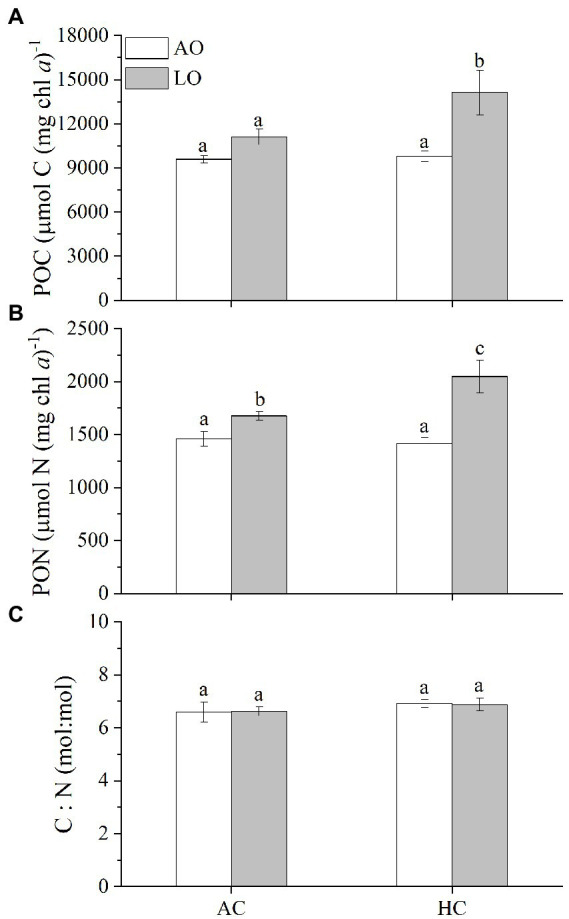
The particulate organic carbon (POC) **(A)**, particulate organic nitrogen (PON) per chl *a* **(B)**, and C:N ratio (mol: mol) **(C)** in *T*. *erythraeum* IMS101 grown and acclimated under the different levels of O_2_ and CO_2_ (see Figure 1) for 36 days (27 generations). The values are indicated as the means ± SD for triplicate cultures at each treatment. Different letters above the bars indicate significant (*p* < 0.05) differences among the treatments.

Decreased O_2_ levels to about 60 μM slightly raised the cellular particulate organic carbon (POC) by 16% (*p* = 0.0583) and particulate organic nitrogen (PON) by 15% (*p* = 0.0190) in the *T. erythraeum* cells ISM 101 grown under the AC (Figures 4A,B). Contrastingly, under the HC conditions, the reduced O_2_ concentration increased POC by 44% (*p* = 0.0002) and PON by 45% (*p* < 0.0001), respectively (Figures 4A,B). Nevertheless, the cellular C:N ratios were about 6.8, being neither affected under the changed levels of *p*CO_2_ (*p* = 0.0796) nor under the reduced levels of *p*O_2_ (*p* = 0.9859) (Figure 4C).

## 4. Discussion

**Figure 5:**
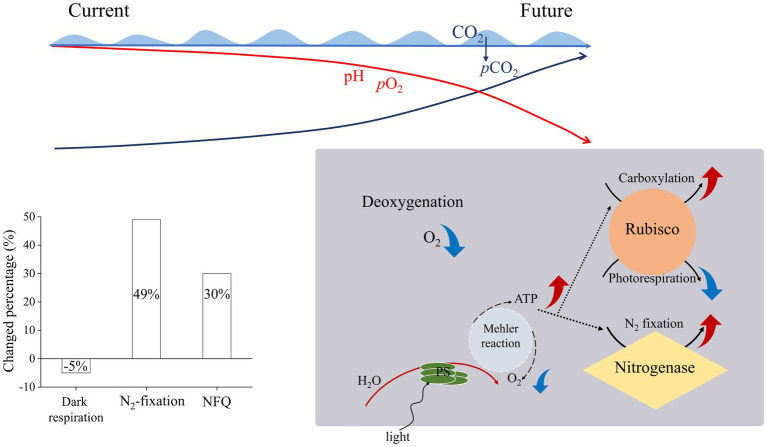
Illustration of the effects of ocean deoxygenation and acidification on *Trichodesmium erythraeum*, which is suggested to decrease its dark respiration by 5% and to increase its N_2_-fixation by 49% and N_2_-fixation quotient (NFQ, ratios of N_2_ fixed per C fixed) by 30% with 16% decline of *p*O_2_ and 138% rise of *p*CO_2_ by the end of this century.

In the present study, the *T. erythraeum* cells acclimated to lowered *p*O_2_ and elevated *p*CO_2_ levels showed higher N_2_-fixation quotient (ratio of N_2_ fixed to gross O_2_ production), being increased by 143% (with *p*O_2_ declined bys 75%) and by 12% (with *p*O_2_ declined by 10%) compared to ambient levels of *p*O_2_ and *p*CO_2_ (Figure 5). This could be attributed to decreased dark respiration and increased photosynthetic performance as well as increased N_2_ fixation under the reduced levels of *p*O_2_ and to increased rates of dark respiration and N_2_ fixation under the elevated *p*CO_2_ (Figures 2, 3). Although both low *p*O_2_ and high *p*CO_2_ treatments did not significantly affect the growth rates of *T. erythraeum*, its cellular particulate organic carbon (POC) and nitrogen (PON) contents increased under the lowered O_2_ and elevated *p*CO_2_ levels. Our data provided the experimental evidence that reduced O_2_ levels increased N_2_ fixation and photosynthetic performance by stimulating the activities of nitrogenase and Rubisco (Figure 5), enhancing POC and PON production in anoxic and/or hypoxic environments, where lowered levels of O_2_ and pH (high *p*CO_2_) are covarying drivers.

### 4.1. Responses of N_2_ fixation, photosynthesis, and growth to deoxygenation

Nitrogenase, the key enzyme that catalyzes the reduction of N_2_, is instantaneously and irreversibly inactivated by O_2_ *via* oxidative damage (Staal et al., 2007). Meanwhile, significantly decreased photosynthetic O_2_ evolution coincided with increased respiratory CO_2_ release during midday, that favored the N_2_ fixation (Kranz et al., 2009). In the present work, reduced levels of O_2_ in milieu significantly increased N_2_-fixation and photosynthesis (Figures 2, 3). Since lowered O_2_ concentration increased the net photosynthetic O_2_ evolution, the dissolved O_2_ in the cultures increased before the continual dilutions, which, nevertheless, did not significantly alter the difference in O_2_ treatments between lowered (LO) and ambient (AO) *p*O_2_ (Table 1). Therefore, the O_2_ levels (AO: 245–255 μM; LO: 60–90 μM) during the growth period could still reflect LO and AO conditions. In photosynthesis, electrons transport *via* the photosynthetic and respiratory transport chains must have promoted production of ATP and reductant for N_2_ fixation and CO_2_ assimilation in *Trichodesmium* (Suggett et al., 2010; Eichner et al., 2019). While no decline in net photosynthetic O_2_ evolution was found with enhanced dark respiration in this study (Figure 2), higher respiration rates during the photoperiod would decrease the photosynthetic O_2_ evolution in *Trichodesmium* (Berman-Frank et al., 2001), which must partially favored the N_2_ fixation. In the present work, the rates of dark respiration accounted for 37% in AO-grown and for 18% in LO-grown cells of the gross photosynthesis (Figures 2B,C), indicating that the role to remove O_2_ by respiration was down-regulated under LO. Since the O_2_ consumptions such as dark respiration were reduced under LO, and the net photosynthesis significantly increased (Figure 2), Mehler reaction must have been active in maintaining low intracellular O_2_ to sustain the activity of the nitrogenase and promote energetically expensive N_2_ fixation. Additionally, it is most likely that the *Trichodesmium* cells grown under low O_2_ could have greater abundances and/or upregulated activity of the nitrogenase (Zehr et al., 1993). Such physiological responses can be responsible for increased values of the N_2_-fixation quotient under the low O_2_ conditions (Figure 3C), indicating that the N_2_-fixation efficiency increased per photosynthetically evolved O_2_ or C fixed under low O_2_ and high CO_2_ conditions.

### 4.2. Combined effects of deoxygenation and acidification

Although the cells of *Trichodesmium* grown under HC increased their N_2_ fixation under both ambient and lowered *p*O_2_ (Figure 3), their specific growth rates were unaffected compared to the AC-grown cells (Figures 1, 2). This contradicts to some of the previously reported results (Table 2), which show either enhanced or inhibited growth rates of *Trichodesmium* grown under future ocean acidification conditions. The likely reason responsible for such discrepancies between these studies (Table 2) could be that different light sources (different emission spectra) regulate the diazotroph’s response to elevated *p*CO_2_ even under equal levels of PAR (Yi and Gao, 2022), though such hypothetical explanation should be based on N_2_-fixation action spectra, which has not been documented. In addition, limitation of phosphate and iron ions as well as exposure to solar UV radiation could also result in negative or insignificant effects of future ocean acidification on the N_2_-fixation of *Trichodesmium* (Hong et al., 2017; Yi and Gao, 2022; Zhang et al., 2022). In the present study, when the *Trichodesmium* cells acclimated to the acidified HC treatment with replete phosphate and iron, its photosynthesis and growth did not show significant changes, though its N_2_-fixation increased by 129% under ambient and by 38% under the reduced *p*O_2_, respectively (Figure 3). In addition, the HC treatment increased its POC significantly under the LO (Figure 4), though its photosynthesis did not show significant change (Figure 2). Such inconsistency between the photosynthetic responses and the POC production could be attributed to altered photosynthetic quotients (ratios of evolved O_2_ to CO_2_ fixed). In the present work, gross photosynthetic rates changed less compared to the net photosynthetic rates, since dark respiration decreased when net photosynthesis increased under the different combinations of *p*O_2_ and *p*CO_2_. On the other hand, the documented photosynthetic quotients of *Trichodesmium* ranged 1.28–2.60 (Kranz et al., 2010; Boatman et al., 2019). Therefore, photosynthetic O_2_ evolution rates can hardly be taken as a proxy of C fixation, which must be responsible for the observed discrepancy between the POC production and photosynthetic O_2_ evolution.

**Table 2:** Documented growth rates (μ) and nitrogen fixation rates (NF) of *T. erythraeum* grown under low (350 ~ 400 μatm) and high (750 ~ 1,400 μatm, values in the brackets) CO_2_ conditions.

References	Strain	Light (μE m^−2^ s^−1^)	T (°C)	μ	% (μ)	NF^	% (NF)	Methods^#^
Hutchins et al. (2007)	GBR	100	25	0.29 (0.36)	24%*	18.3 (26.1)	43%*	ARA
	IMS101	100	25	0.35 (0.39)	11%	14.8 (20.0)	35%*	
Levitan et al. (2007)	IMS101	80–120	25	0.17 (0.26)	53%*	5.6 (16.8)	200%*	ARA
Kranz et al. (2010)	IMS101	200	25	0.39 (0.43)	11%*	7.6 (16.7)	120%*	ARA
Levitan et al. (2010)	IMS101	80	25	0.17 (0.33)	94%*	4.4 (20.0)	355%*	ARA
Garcia et al. (2011)	IMS101	100	27	0.22 (0.31)	41%*	12.6 (15.1)	20%	^15^N
		220	27	0.27 (0.38)	41%*	18.1 (23.8)	31%*	^15^N
Shi et al. (2012)	IMS101	90	27	0.26 (0.19)	−27%*	2.5 (1.6)	–34%*	^15^N
			27	0.46 (0.37)	−20%*	3.4 (1.7)	−50%*	^15^N
Eichner et al. (2014)	IMS101	150	25	0.34 (0.31)	−9%*	6.2 (10.6)	71%*	ARA
Hutchins et al. (2015)	IMS101	120	26	0.25 (0.36)	44%*	13.0 (18.6)	43%*	ARA
Hong et al. (2017)	IMS101	80	27	0.57 (0.47)	−18%*	13.1 (10.6)	−19%*	ARA
Zhang et al. (2022)	IMS101	80	27	0.2 (0.2)	—	7.6 (5.3)	−30%*	^15^N
Yi and Gao (2022)	IMS101	sunlight	25	0.16 (0.14)	−12%	4.7 (4.6)	−1%	ARA
This study	IMS101	160	27	0.53 (0.52)	−2%	8.4 (19.2)	129%*	ARA

“%” represents the percentage of decrease (−) or increase due to the elevated *p*CO_2_.

The values marked with “*” indicate significant change (*p* < 0.05).

^ N2 fixation rates per h−1 based either on chl a or cell number.

# acetylene reduction method (ARA) or ^15^N_2_ isotope tracer method for N_2_ fixation measurement.

Carbon and nitrogen fixation are both energy-intensive processes that compete directly for the products of photosynthesis (Berman-Frank et al., 2001; Hutchins et al., 2007). Previously, the enhancement of N_2_ fixation as well as growth rate of *Trichodesmium* at elevated *p*CO_2_ levels has been considered as an indirect effect resulting from alleviation of C-limitation of CO_2_ fixation (Hutchins et al., 2007) or attributed to saved energy from down-regulation of CCMs (Table 2). However, Shi et al. (2012) and Hong et al. (2017) showed that the N_2_ fixation rate of *T. erythraeum* significantly decreased under the elevated *p*CO_2_ projected for future acidification, which was attributed to reduced efficiency of nitrogenase, although the expression of the nitrogenase was enhanced in terms of proteomic responses (Zhang et al., 2019; Wen et al., 2022). Our data did not show significant effects of the elevated *p*CO_2_ on the growth rate and photosynthesis in *T. erythraeum*, but its N_2_ fixation rate and POC/PON were enhanced (Figures 2–4). It is likely that the energy saved from downregulation of CMMs under HC was not enough to bring significant increase of net photosynthesis and growth rate (Luo et al., 2019). Considering that phytoplankton can benefit from the elevated *p*CO_2_ under light but suffer from the acidic stress in darkness, resulting in daytime enhanced and nighttime suppressed growth rates (Qu et al., 2021), the net effects of the HC treatment on growth should be holistically considered for the diel cycle and availability of nutrients (Table 2). We suggest that extra energy required to cope with the acidic stress under HC could be provided *via* increased dark respiration (Figure 2), compensating for the insufficient energy supply from the downregulation of CCMs in *Trichodesmium* cells grown under HC. In the present work, lowered levels of O_2_ enhanced photosynthesis and POC production of *Trichodesmium* (Figures 2, 4). This could be attributed to suppressed respiration (Figure 2), which enables the cells to save more POC (Figure 4A).

### 4.3. Discrepancy between the N_2_ fixation and PON production

Since one time point measurement might have overlooked daily N_2_ fixation, the rates of N_2_ fixation in this work could hardly be approximated to that of PON production (Figures 3, 4). When the N_2_ fixation rate was integrated for the daytime, it turned out that PON production rate (Supplementary Figure S3) was higher than the integrated N_2_ fixation per day, which was about one third of the PON production rates. Similar discrepancy was also reported by Zhang et al. (2019), whose daily PON production rate was about 3 times the daily integrated N_2_ fixation. In the present work, increased proportion (15%) of PON is much less than the enhancement (139%) of N_2_ fixation under LO compared to AO under ambient CO_2_ concentration (Figures 3, 4), such inconsistency could be contributed to the fact that PON production reflects how much new fixed N is ultimately incorporated into the cells which covers several generations, while N_2_ fixation rate measured within 2 h during the midday could only reflect the periodical performance during light period. Future studies should examine the diurnal variations of the N_2_ fixation to look into the diel or diurnal responses. Meanwhile, increased availability of CO_2_ (Kranz et al., 2010; Hutchins et al., 2015) and elongated light period (Cai and Gao, 2015) could alter the maximal rate of N_2_ fixation from the mid-light period, the low O_2_ treatment might have led to different levels of N_2_ fixation rates at different time points during the light period, which could also be responsible for the inconsistency. On the other hand, since the cells can release 10 to 50% of the fixed N as dissolved organic N (DON) and / or NH_4_^+^ into seawater (Mulholland et al., 2004; Konno et al., 2010; Lu et al., 2018), the proportional increase of PON could be less. The exudation of “new-fixed” N may be further increased at elevated *p*CO_2_ (Hutchins et al., 2007), which is mirrored in our study in that the elevated *p*CO_2_ enhanced the N_2_ fixation but not changed the cellular PON under the ambient O_2_ level (Figures 3, 4).

### 4.4. Implications for future ocean deoxygenation and acidification

The global oxygen loss has been suggested to be about 2% of the total ocean inventory per decade since 1960 (Schmidtko et al., 2017) and the O_2_ concentration of ocean has been predicted to decline to about 200 μmol L^−1^ by the end of the century (−5 μmol kg^−1^ per decade) (Breitburg et al., 2018). In addition, upwelling-induced hypoxia events have been shown in sunlit layers in time-series observations (Gireeshkumar et al., 2017), and typhon-driven mixing would churn deep seawater of low O_2_ and high CO_2_ to surface layer. Therefore, diazotrophs and other phytoplankton are inevitably exposed to extreme low O_2_ and high CO_2_ conditions. This study provided the experimental evidence that combination of lowered *p*O_2_ and pH along with elevated *p*CO_2_ enhanced the photosynthesis and N_2_-fixation of *Trichodesmium*. Under the future scenarios of ocean deoxygenation and acidification, as estimated in the present work, *Trichodesmium* would decrease its dark respiration by 5% and increase its N_2_-fixation by 49% and N_2_-fixation quotient by 30% with 16% decline of *p*O_2_ and 138% rise of *p*CO_2_ by the end of this century (Supplementary Table S1; Figure 5). Such estimation might overestimate the changed percentage, since increased POC and PON only accounted for 10% and by 8%, respectively (Figure 4). As increased temperature would also enhance iron use efficiency (Jiang et al., 2018) and stimulate dark respiration and then favors N_2_-fixation, decreased O_2_ availability along with ocean warming can further enhance the activity of the nitrogenase and increased N_2_-fixation of *Trichodesmium*, as long as ocean warming does not surpass the thermal tipping point for its growth.

## Data availability statement

The raw data supporting the conclusions of this article will be made available by the authors, without undue reservation.

## Author contributions

KG and HL designed the experiment, and analyzed the data and wrote and improved the manuscript. HL carried out the experiment. All authors contributed to the article and approved the submitted version.

## Funding

This study was supported by National Natural Science Foundation of China (nos. 41721005, 41720104005, and 41890803).

## Conflict of interest

The authors declare that the research was conducted in the absence of any commercial or financial relationships that could be construed as a potential conflict of interest.

## Publisher’s note

All claims expressed in this article are solely those of the authors and do not necessarily represent those of their affiliated organizations, or those of the publisher, the editors and the reviewers. Any product that may be evaluated in this article, or claim that may be made by its manufacturer, is not guaranteed or endorsed by the publisher.
