# Supplementary material for: Deoxygenation enhances photosynthetic performance and increases N2 fixation in the marine cyanobacterium Trichodesmium under elevated pCO2
Source: Front Microbiol. 2023 Feb 15;14:1102909. doi: 10.3389/fmicb.2023.1102909 (PMC9975739; doi:10.3389/fmicb.2023.1102909)
Supplement: Supplementary file 1 [file Data_Sheet_1.docx]

Supplementary Materials for

Deoxygenation enhances photosynthetic performance and increases N_2_ fixation in the marine cyanobacterium *Trichodesmium* under elevated *p*CO_2_

He Li^1^, Kunshan Gao^1,2*^

^1^ State Key Laboratory of Marine Environmental Science, College of Ocean and Earth Sciences, Xiamen University, Xiamen 361102, China

^2^ Co-Innovation Center of Jiangsu Marine Bio-industry Technology, Jiangsu Ocean University, Lianyungang 222000, China

*e-mail: [ksgao@xmu.edu.cn](mailto:ksgao@xmu.edu.cn)

**Supplementary information**

**Fig. S1** Chl *a* concentrations between the dilution intervals during the *T. erythraeum* IMS101 culture experiments and timing points at which the physiological and biochemical parameters were measured. Cells acclimated under (A) ambient CO_2_ & ambient O_2_ (black circles), (B) ambient CO_2_ & low O_2_ (red circles), (C) high CO_2_ & ambient O_2_ (black triangles) and (D) high CO_2_ & low O_2_ (red triangles). Lines represent chl *a* concentration in the culture system (before and after diluting the medium every 48 h with newly prepared medium at the target CO_2_-O_2_ levels). MR, NP and PR represent dark respiration, net photosynthetic O_2_ evolution and photorespiration; NF, N_2_-fixation; C/N, particular organic carbon to nitrogen ratios; μ, specific growth rate. All the measurements were carried out after the cells had acclimated for at least 20 generations.

**Fig. S2** Dissolved O_2_ **(A)** and pH_NBS_ **(B)** during the *T. erythraeum* IMS101 culture studies. Symbols of different colors and shapes are the same as in Supplementary Fig. 1 (red indicate low O_2_). The dissolved O_2_ concentrations varied less than 10 μM and 30 μM under AO and LO treatments, respectively. The pH variations were less than 0.08 units under either the HC or AC treatments.

**Fig. S3** The particulate organic carbon (POC) (A) and particulate organic nitrogen (PON) (B) production rates in *T*. *erythraeum* IMS101 grown and acclimated under the different levels of O_2_ and CO_2_ (see Fig. 1) for 36 days (27 generations). The production rates of POC or PON were calculated by multiplying the cellular quota by corresponding specific growth rates (d^-1^). The values are indicated as the means + SD for triplicate cultures at each treatment. Different letters above the bars indicate significant (*p* < 0.05) differences among the treatments.

**Table S1** Estimated changes of respiration, net photosynthesis, N_2_-fixation and N_2_ fixation quotient (N_2_ fixed per O_2_ produced) based on the values in Figs 2, 3 and assuming that these rates in the cells grown at the elevated *p*CO_2_ increases linearly with declined *p*O_2_ as reflected in the cells grown at the ambient *p*CO_2_.

|  | Dark respiration | Net O_2_ evolution | N_2_-Fixation | NFQ |
| --- | --- | --- | --- | --- |
| ACLO | -9 % | 14 % | 14 % | 19 % |
| HCLO | -5 % | 16 % | 49% | 30 % |
